# Mental Health Exploration and Variables Associated with Young Health Professionals in Early Childhood Care Centers: A Systematic Review

**DOI:** 10.3390/healthcare13182354

**Published:** 2025-09-18

**Authors:** Sofía Gómez-Herrera, María Auxiliadora Robles-Bello, David Sánchez-Teruel

**Affiliations:** 1 Faculty of Humanities and Educational Sciences, University of Jaen, 23071 Jaen, Spain; sgh00013@red.ujaen.es (S.G.-H.); marobles@ujaen.es (M.A.R.-B.); 2 Faculty of Psychology, University of Granada, 18011 Granada, Spain

**Keywords:** mental health, resilience, empathy, healthcare professionals, depression, anxiety, burnout

## Abstract

Early childhood care (ECC) represents a vital service for the families two supports; however, research on the experiences of young professionals working in this field is lacking. The nature of the work is inherently difficult due to lengthy bureaucratic procedures, limited flexibility to adapt services to individual needs, and a lack of financial and human resources. **Background/Objectives**: This systematic review is to analyze the existence of scientific literature related to mental health and protective and risk factors in these young professionals. **Methods**: The PRISMA methodology and a comparative analysis of the selected articles were used, incorporating sources from major scientific databases, such as Scopus, Web of Science, and PsycInfo. **Results**: A total of 19,943 articles were identified, with a striking 0% specifically addressing early childhood intervention. Only 13 of the articles were selected for the analysis of mental health among young healthcare professionals. **Conclusions**: The literature reviewed highlights risk factors such as depression, anxiety, and stress among health professionals, as well as protective factors like resilience, social support, empathy and the working conditions themselves (working method, working hours, pay, professional value of the workers themselves, administrative workload and opportunities for teamwork). This study is valuable for establishing a scientific foundation for this population and for enhancing its positive characteristics.

## 1. Introduction

Mental health is increasingly recognized as a key component of societal well-being. The World Health Organization (WHO) [1] defines it as a state of well-being in which individuals are aware of their own abilities, can cope effectively with the normal stresses of life, are productive, and able to contribute to their community. In recent years, mental health has emerged as a pressing social, economic, and political concern across countries worldwide. According to the Pan American Health Organization (PAHO) in its World Mental Health Report, one in eight people worldwide suffers from a mental disorder—the most common being depressive and anxiety disorders—posing a significant burden on public health systems. Despite the prevalence and impact of these conditions, national budgets allocate, on average, less than 2% of their resources to mental health [2]. This concern is particularly salient in contexts such as early childhood care centers (ECC), where professionals play a crucial role in child development. Nevertheless, the mental health of these professionals has been scarcely investigated, highlighting a significant empirical gap [3].

Mental health among healthcare professionals has increasingly become a focal point of research [4], with growing concern regarding its impact on both the well-being of the professionals themselves and the quality of care they provide. Healthcare professionals, especially those working in high-stress environments, such as ECC, are particularly vulnerable to burnout. This condition leads to a decline in their emotional well-being and productivity, which, in turn, results in poor patient care and higher staff turnover [5,6,7]. It is important to note that the factors contributing to work stress and burnout are exacerbated for mental health professionals, as additional elements come into play, such as the stigma surrounding mental health, exposure to negative emotions, dealing with unstable patients, managing suicidal thoughts, and the constant demands of documentation, among others [8].

Within the field of ECC, multiple factors contribute to the progressive deterioration of professionals’ emotional well-being. Among the most prominent are highly demanding working conditions, including excessive bureaucratic procedures, limited flexibility in adapting resources to the individual needs of children, and insufficient financial and human resources [9]. From a general perspective, professionals in early childhood care devote a considerable portion of their workday to administrative responsibilities, which constrains the time available for intervention planning, detailed case documentation, and interdisciplinary collaboration [10]. These structural constraints not only hinder service delivery, but also undermine the quality of care provided to children and their families [11]. Families, for instance, often experience significant psychological distress upon receiving the news that their child may have a developmental disability [12,13]. In addition to providing support to children with severe conditions, professionals are also required to manage the emotional burden experienced by families—particularly mothers—who are confronting the implications of such diagnoses [3,14,15]. Evidence further suggests that professionals with less experience in the field tend to report higher levels of perceived stress compared to their more experienced counterparts [16]. They also exhibit lower tolerance for uncertainty, greater apprehension about making errors, and employ coping strategies that are less effective than those of more experienced professionals [17]. It is paramount to recognize that the support provided by early childhood intervention professionals to families constitutes a critical determinant in shaping early developmental outcomes [18]. Consequently, it is crucial to investigate the mental health of professionals working in ECC settings, as well as the protective factors that may buffer the impact of these occupational challenges.

Several factors have been identified in the literature as key contributors to the mental health of healthcare professionals. One of the most significant of these is resilience, which has been proposed as a key factor in reducing burnout, while also empowering professionals by enhancing self-confidence and self-regulation [19]. Empirical evidence indicates that higher levels of emotional self-regulation are associated with greater job satisfaction and lower burnout [20], while professionals with elevated emotional intelligence tend to employ more adaptive coping strategies in highly demanding occupational contexts [21]. In this regard, resilience acts as a self-regulatory mechanism that shields individuals from the negative impact of stressful life events [22]. Furthermore, it has been argued that a competent, motivated, and resilient workforce is necessary to achieve the sector’s objectives [23,24]. Within this framework, emotional intelligence plays a crucial role by fostering empathy, compassion and self-care, factors that enhance both professional well-being and the quality of patient care [25]. These findings are consistent with those of Mealer [26], who demonstrated that healthcare professionals with higher resilience levels were better equipped to manage occupational stress and prevent burnout. However, various work-related factors have been identified that reduce the performance and life satisfaction of healthcare professionals, such as emotional stress [27].

Similarly, self-efficacy is considered a key factor in strengthening resilience. As defined by Bandura [28], self-efficacy refers to an individual’s belief in their capacity to cope with potentially stressful situations, which in turn guides the organization and execution of behaviors aimed at achieving desired outcomes [29]. Research has shown a negative correlation between self-efficacy and psychological distress [30]. Several studies highlight its buffering role against work-related stressors, such as excessive working hours, task overload, time pressure or routine demands [31,32,33]. A high sense of self-efficacy allows a person to view difficult tasks as transformative rather than threatening, thus increasing goal attainment, reducing stress, and lowering the likelihood of depression. In contrast, a person with low self-efficacy tends to experience deficits in productivity and results, alongside a reduced likelihood of personal development [34,35].

In this context, some researchers have established a link between resilience and emotional intelligence (EI), suggesting that individuals with higher levels of emotional intelligence exhibit greater resilience, adapt more effectively to change under stressful conditions, and perceive stress as a challenge rather than a threat [36]. Defined as the ability to perceive, understand, and regulate one’s own and others’ emotions [37], EI is associated with greater professional success, job satisfaction, and more effective coping strategies, particularly in high-demand settings, such as healthcare [38,39]. Within EI, empathy enable individuals to comprehend others’ emotions and regulate their own, thereby facilitating social and professional adaptation. In healthcare contexts, this skill contributes to enhancing patient experience and strengthening the therapeutic relationship. For instance, a study by Chaitoff concluded that higher levels of empathy among professionals were associated with improved patient care experiences [40,41]. This notion is further substantiated by the qualitative study conducted by Guillot-Valdés [42], wherein families of children with disabilities receiving services at ECC underscored the critical role of professional empathy in reinforcing the therapeutic alliance and promoting optimal developmental outcomes in the child.

Another significant protective factor is social support, which encompasses the material, cognitive, and affective resources that professionals receive from various sources (e.g., family, friends, colleagues, supervisors) to cope with occupational stress [43,44]. Professionals who perceive this support tend to experience assertive communication with colleagues and supervisors, receive constructive feedback that acknowledges their work, and benefit from guidance in managing stressful situations. The absence of support, particularly from supervisors, has been linked to increased risk of burnout, whereas its presence correlates with higher job satisfaction and better emotional self-regulation in the workplace [45,46]. Consequently, these elements are crucial for cultivating a healthier and more positive work environment.

However, research in this specific area remains notably scarce and warrants further exploration, as its neglect may hinder the developmental progress of service users [3,47]. Therefore, the aim of this study was to conduct a systematic review of risk and protective factors that promote resilient outcomes in young health professionals working in early childhood care centers, while examining how these factors have been empirically assessed in the included studies.

## 2. Materials and Methods

### 2.1. Resources and Research Strategies

The review followed a rigorous and transparent process to identify and synthesize existing studies that summarize the available evidence on this subject. Furthermore, a flow diagram was developed in accordance with PRISMA (New York, NY, USA) guidelines [48], to clearly illustrate the selection process and highlight the existing gaps in the literature within the field of early childhood intervention [3]. This review project was registered in PROSPERO (UK) at https://www.crd.york.ac.uk/PROSPERO/view/CRD420251114727, accessed on 12 June 2025.

The databases selected for this systematic review they were consulted between 18 and 30 March 2025, specifically—Scopus (ELSEVIER, Amsterdam, The Netherlands), Web of Science, Clarivate, London, UK), and APA PsycInfo (American Psychological Association, Washington, DC, USA)—were chosen due to their global prominence and comprehensive coverage of high-impact journals in the health, social, and behavioral sciences. Their inclusion ensured an extensive and rigorous retrieval of empirical studies relevant to resilience, emotional intelligence, self-efficacy, and social support among early-career professionals in early childhood care. An advanced search strategy was employed, using the following search terms: “healthcare professionals” AND “empathy”; “healthcare professionals” AND “resilience”; “healthcare professionals” AND “emotional intelligence”; “healthcare professionals” AND “self-efficacy”; “healthcare professionals” AND “social support”; “healthcare professionals” AND “anxiety”; “healthcare professionals” AND “depression”. The descriptor “early childhood intervention” was added to all search combinations to ensure the relevance of the results to the target population.

### 2.2. Eligibility Criteria

The selection process for the publications began with the establishment of a screening procedure, in which inclusion and exclusion criteria were defined and applied. These criteria are detailed in Table 1.

### 2.3. Study Selection Procedure

An initial search using the selected descriptors yielded 19,943 articles across the three databases used in this review. It is important to note that when the keyword “Early Childhood Intervention” was added to the search, no articles were found in any of the databases, which underscores the contribution of Robles-Bello and Sánchez-Teruel [3], who highlight the scarcity of research in the field of early childhood care and its young professionals. In light of these findings, the existing literature on healthcare and young mental health professionals (early-career professionals) will be analyzed.

After applying the established eligibility criteria, 18,841 articles were excluded, leaving 1102 for preliminary screening. Titles were carefully reviewed, and duplicates were meticulously identified and removed through an extensive manual checking in MS Excel, yielding a refined set of 116 studies. Their abstracts were then examined, resulting in 29 articles deemed potentially relevant.

The full texts of these 29 articles were then meticulously evaluated with respect to their objectives, findings, and methodological rigor. A total of 13 articles were ultimately included in the systematic review.

Data extraction was performed independently by a single reviewer. No attempts were made to contact the original study authors for clarification or to obtain additional information. The data collected were synthesized and organized using Microsoft Excel.

### 2.4. Data Analysis

**Figure 1:**
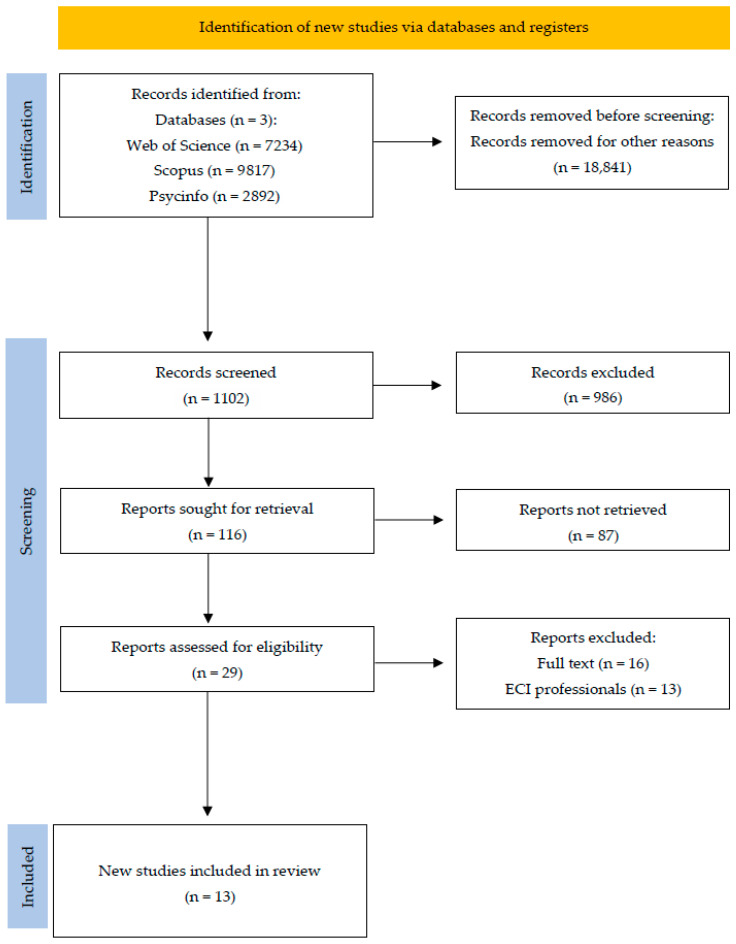
Flow diagram.

To carry out this systematic review, the PRISMA methodology was employed (illustrated in Figure 1), with the objective of analyzing the available scientific studies that examine the relationship between mental health (anxiety and depression) and early childhood health professionals, as well as the relationship between protective variables (emotional intelligence, resilience, social support, and self-efficacy) and early childhood professionals. A comparative analysis of the selected articles was also conducted. The variables identified for this analysis include (1) author; (2) year of publication; (3) participants; (4) methodology; (5) instruments used; and (6) relevant conclusions.

### 2.5. Study Risk of Bias Assessment

The methodological quality and risk of bias of the studies included in this systematic review were independently evaluated using the Newcastle–Ottawa Scale (NOS) [49], a validated instrument for assessing nonrandomized observational studies. This tool examines key domains of study quality, including selection of participants, comparability of study groups, and outcome assessment.

Each study was scored according to the NOS checklist, yielding a maximum score of nine points. Based on the total scores, studies were classified as high quality (7–9 points), moderate quality (4–6 points), or low quality (≤3 points). The assessment was conducted by two reviewers independently, with discrepancies resolved by consensus to ensure objectivity and rigor.

The results of this risk of bias evaluation are summarized in Table 2. Most studies were rated as high quality (*n* = 5), with the remainder classified as moderate quality (*n* = 8). No studies were considered low quality. All included studies employed validated measurement instruments and provided sufficient methodological detail to warrant their inclusion.

This review exclusively included quantitative observational studies that employed validated and standardized questionnaires, without any intervention or experimental manipulation.

## 3. Results

This systematic review includes 13 articles analyzing the mental health of healthcare professionals across different specialties and settings. Although the target population of this research is young professionals working in early childhood care, no specific studies focusing on this group were found. Additionally, none of the articles examined the relationship between emotional intelligence and healthcare professionals.

Table 3 presents a detailed descriptive synthesis of the main characteristics of the studies included in this review. Most of the studies employed quantitative methods, with one using a mixed-methods design. The participants varied: 53.80% involved healthcare professionals in general, 23.10% focused on palliative care professionals, 15.40% on pharmacists, and 7.70% on nursing staff. The table also specifies the variables assessed in each study. Predictors and outcomes are distinguished to clarify the relationships observed. It should be emphasized that no studies have been identified examining emotional intelligence as a factor associated with the resilience of healthcare professionals.

Regarding geographic distribution, 30.80% of the studies were conducted in upper-middle income level (Saudi Arabia, South Africa, and Lebanon) [63]. This information, along with a quantitative summary of the included articles, is presented in Table 4. Most articles (69.20%) were published from 2022 onwards, and all were published in English.

## 4. Discussion

The primary objective of this research was to examine the existence of scientific literature published between 2014 and 2024 concerning mental health and protective factors among healthcare professionals working in early childhood care centers. Specifically, the study aimed to explore the relationship between these professionals and mental health (anxiety and depression), as well as the association between protective factors (resilience, empathy, emotional intelligence, self-efficacy, and social support) and their mental well-being. However, following a comprehensive literature search focused on the personal characteristics of this specific population, no studies were identified that examined the variables of interest among early intervention professionals, as previously noted by Robles-Bello and Sánchez-Teruel [3]. As a result, the scientific articles selected for this review addressed these variables within the broader population of healthcare professionals.

Research in this area over the past decade remains limited; however, it is noteworthy that the majority of the selected articles were published during or after the global COVID-19 pandemic. Several of these studies highlight the impact of the pandemic experience as an influential factor on the personal characteristics of healthcare professionals. The emotional well-being of healthcare professionals received heightened attention during this period, although it is a concern that has endured over time [4].

Regarding the relationship between healthcare professionals and mental health, evidence suggests that this occupational sector is associated with an increased risk of depression. Alwhaibi [51] linked depression to a heightened risk of emotional impairment, depersonalization, and reduced self-fulfillment, which in turn increases the likelihood of burnout. This connection implies that depression may be closely related to burnout in the workplace. Indeed, most studies investigating the mental health of healthcare professionals consider burnout a major risk factor affecting emotional well-being in this field. Ridremont [60], for instance, identified four psychological profiles among healthcare professionals, one of which was the “burnout profile,” characterized by high levels of stress and low perceived rewards, potentially leading to diminished quality of patient care.

Similarly, stress and anxiety have been shown to negatively impact both patient care and the emotional well-being of healthcare professionals. These factors are closely linked to burnout, which in turn increases the risk of depression, reduces empathy, and impairs communication abilities [55]. Several studies have examined the association between empathy and these psychological variables, generally finding a negative relationship. Specifically, high levels of anxiety may diminish the warmth and engagement with which patients are treated and may adversely affect social skills and openness to change, thereby increasing susceptibility to psychological burnout [53].

The empirical literature has identified several protective factors against emotional distress in healthcare professionals. Firstly, Weiss [62] highlights resilience as a significant negative predictor of burnout; that is, individuals with high levels of resilience are more likely to maintain emotional stability and adapt effectively to adversity, thereby reducing the risk of developing burnout. Numerous studies support this conclusion, with some even suggesting that resilience alone can mitigate symptoms of depression and anxiety [51,56].

Resilience is conceptualized as a personal resource that allows healthcare professionals to maintain well-being and professional efficacy in demanding work contexts. According to Maffoni [58], resilience exerts its protective role through the mediation of an ethical vision of patient care and the moderation of managerial support, suggesting that resilient individuals are better able to sustain their professional effectiveness while promoting quality patient care. Consequently, resilience is highlighted as a key factor safeguarding the emotional and occupational functioning of healthcare professionals.

The relationship between empathy and burnout has also been explored in this field. This syndrome is understood as a defense mechanism to cope with the experiences of patient suffering and death to which healthcare professionals are exposed [50,54,57], leading them to disengage emotionally and act in the opposite way to empathetically. However, factors derived from empathy, such as perspective-taking and empathic concern, have been found to be unrelated to burnout [55]. Despite this, healthcare professionals require emotional stability and perceived social support from their immediate environment, particularly after adverse events at work, as the lack of such support can reduce their perceived self-efficacy. Social support has been studied as a facilitator of positive physical and mental health, with particular emphasis on support from supervisors, even though it may not necessarily contribute to increasing employees’ own self-efficacy [52,54,58,59].

Factors that can enhance the resilience of healthcare workers have also been assessed, with key elements including working methods, working hours, pay, the professional value of the workers themselves, administrative workload, and opportunities for teamwork, among others. These conditions can foster a positive organizational climate, where staff feel secure in their roles, thereby contributing to the maintenance of positive mental health. This, in turn, leads to improved quality of care for the service beneficiaries [60,61].

### 4.1. Clinical Implications for Young Professionals in Early Childhood Care

The findings of this study hold significant implications for both professional practice and policy development within the health sector. Importantly, these results underscore the necessity of opening new avenues of research in early childhood care, with particular emphasis on young professionals entering this field. This review highlights the paucity of the literature available for this sector, as no studies on ECC were identified. Furthermore, based on the evidence examined, it can be recommended that ECC implement structured training and support programs aimed at fostering self-care strategies among professionals, especially those at the early stages of their careers. Such initiatives should include the cultivation of protective factors, including resilience, self-efficacy, and empathy. The promotion of these personal resources may contribute not only to enhancing the emotional well-being of young professionals, but also to improving the overall quality of care provided to service users.

Simultaneously, it is essential to foster a supportive organizational climate that facilitates teamwork, assertive communication, and perceived social support, particularly from supervisors. These organizational conditions have been identified as protective elements that may prevent burnout and enhance job performance within this professional group.

Future research should specifically focus on identifying risk and protective factors among young professionals working in early childhood care centers, with the inclusion of emotional intelligence among these factors. Such efforts would contribute to the design and implementation of targeted, evidence-based interventions that support the mental health of these professionals and the sustainability of the services they provide.

### 4.2. Limitations

This systematic review is subject to several limitations that must be acknowledged. Firstly, there is a notable absence of empirical studies specifically addressing the mental health of young professionals working in the field of early childhood care. This scarcity reflects the still incipient and limited consolidation of this area of research, which restricts both the generalizability and the applicability of the findings to this professional group, whose relevance is critical for strengthening more inclusive and sustainable care systems.

Secondly, a temporal bias must be noted, as a considerable proportion of the included studies were conducted during or immediately after the COVID-19 pandemic. This extraordinary context may have influenced the mental health indicators and protective factors under examination, thereby limiting the extrapolation of results to other historical or social circumstances.

Additionally, a lack of homogeneity in the definition of the “young professional” category was observed. The absence of a consensual and consistent age range across the reviewed studies hinders meaningful comparisons and provides only a partial and fragmented understanding of this variable, ultimately reducing the robustness of the conclusions that can be drawn.

Another relevant limitation concerns the linguistic criterion applied in the selection of articles, as only studies published in English or Spanish were included. While this decision was methodologically necessary to ensure the rigorous assessment of the texts, it may have excluded relevant research published in other languages, thus restricting the breadth and diversity of the knowledge considered.

Finally, a future research agenda is necessary, aiming at overcoming these limitations. Although the available evidence supports certain associations in general healthcare contexts, a substantial gap persists within the domain of early childhood care. In this regard, it will be essential to promote cross-sectional and longitudinal studies employing validated instruments to design organizational interventions—such as strengthening supervisor support and reducing administrative burdens—and to develop controlled trials of programs aimed at enhancing resilience and self-efficacy, specifically adapted to the needs of ECC professionals.

## 5. Conclusions

This study has focused attention on the mental health of young early childhood care professionals, revealing a significant knowledge gap regarding public policies for early childhood care, and especially regarding the mental health of health professionals new to this field or those who have only recently begun working in it. This gap has been highlighted in this review. Specifically, significant limitations have been detected on the part of public and private administrations in implementing psychological hygiene programs for young professionals exposed to high levels of professional demands. However, there are also viable solutions that should be assessed by the relevant administrations regarding the implementation of protocols to promote factors that foster high levels of resilience.

Finally, it is important to highlight the push to improve the development of factors that protect against stress, anxiety, and burnout in young professionals in this field. This will increase the efficiency and effectiveness of programs applied to children at an early age, their families, and their environment. This could lay the groundwork for greater universalization, free access, and quality of this service, as well as promoting increased sustainability over time.

## Figures and Tables

**Table 1:** Eligibility criteria.

Criteria	Inclusion	Exclusion
Date of publication	2014–2024	Before 2014 or after April 2024
Type of document	Quantitative research articles	Gray literature (websites, blogs) and review articles
Research area	Healthcare, mental health professionals, and young mental health professionals	Non-healthcare areas
Language	Articles in English and/or Spanish	Articles written in any language other than English and/or Spanish
Article focus	Articles focused on analyzing the relationship between mental health and healthcare professionals and/or the relationship between protective variables and healthcare professional	Articles that do not aim to analyze the relationship between mental health and protective factors in health professionals

**Table 2:** Quality scores of cross-sectional studies (Newcastle-Ottawa scale).

Study	Selection	Comparability	Exposure	Total Quality Score
Alameddine [50]	2	2	2	6
Alwhaibi [51]	3	2	2	7
Alyahya and AboGazalah [52]	3	2	2	7
Ayuso-Murillo [53]	2	1	2	5
Delafontaine [54]	3	2	2	7
Hunt [55]	3	2	2	7
Jackson [56]	2	1	2	5
Johnson [57]	4	2	3	9
Maffoni [58]	2	2	2	6
Mathebula [59]	2	2	2	6
Ridremont [60]	2	2	2	6
de Almeida [61]	2	2	2	6
Weiss [62]	2	2	2	6

**Table 3:** Descriptive summary of study features and assessed variables.

Author(s)	Year	Variables Studied	Participants	Methodology	Instruments Used	Relevant Conclusions
Alameddine [50]	2022	Predictor: resilience; outcome: burnout, job satisfaction, turnover intention, workload, pay, perception of safety	Pharmacists	Quantitative	CD-RISC; CBI; ad hoc	Lower levels of resilience are associated with higher levels of burnout
Alwhaibi [51]	2022	Correlational: depression, socio-demographic variables and burnout	Health professionals in hospital	Quantitative	PRIME-MD; MBI; ad hoc	Depression is characterized by burnout symptoms in the occupational setting. Interventions are needed to improve the mental health of healthcare professionals
Alyahya and AboGazalah [52]	2021	Predictor: stress; outcome: social support, work role conflict, work role ambiguity, work overload	Health professionals in primary care centers	Quantitative	PSS; MSPSS; the role ambiguity and the role conflict measure	Social support is associated with stress
Ayuso-Murillo [53]	2020	Correlational: empathy and anxiety	Nursing professionals in public hospitals	Quantitative	16PF-5; ad hoc	Higher levels of anxiety negatively impact warmth, liveliness, social skills, and openness to change—symptoms that are closely associated with burnout
Delafontaine [54]	2024	Predictors: personality variables, meaning at work; outcome: anxiety, depression, burnout, well-being	Oncology and palliative care health professionals	Quantitative	MBI-HSS; IPWW; HADS; RSE; MS; TIPI; CSDS	Burnout is considered a defense mechanism against suffering and experiences related to death, and is often regarded as the antithesis of empathy
Hunt [55]	2019	Correlational: compassion satisfaction, secondary traumatic stress and burnout	Health professionals involved in the care of people with cancer	Quantitative	ProQOL-V; IRI; ad hoc	Higher stress levels are associated with increased burnout and secondary traumatic stress, leading to greater depression, reduced empathy, and poor communication
Jackson [56]	2024	Predictors: anxiety, depression and resilience; outcome: burnout	Critical care health professionals	Quantitative	Mini-Z; PHQ-9; GAD-7; BRS-J; ad hoc	Resilience decreases with depressive and anxious symptoms. Strengthening resilience, teamwork, and safety reduces burnout
Johnson [57]	2015	Predictor: resilience training program; outcome: depression, anxiety, perceived stress	Depressed health professionals	Quantitative	Intervention program	Enhancing resilience contributes to reduced depressive symptoms
Maffoni [58]	2022	Predictor: resilience; outcome: well-being, professional self-efficacy, ethical vision of patient care, managerial support	Health professionals involved in neuro-rehabilitation or palliative care	Quantitative	CD-RISC; MASI-R; MBI; ad hoc	Resilience shows a positive association with well-being and self-efficacy, contributing to improved quality of care
Mathebula [59]	2022	Correlational: second victim experience, stress, social support, self-efficacy, turnover intentions, absenteeism	Hospital professionals (including administrative staff)	Quantitative	SVEST	Professionals require active involvement from their supervisors. Adverse events are linked to decreased self-efficacy, increased turnover intentions, absenteeism, and stress symptoms
Ridremont [60]	2024	Correlational: burnout, work rewards, coping strategies, socio-demographic variables, quality of patient care	Health professionals (medicine, nursing and auxiliary nursing) dedicated to child and adolescent cancer care.	Quantitative	PCSQ; WRS-PO; WCC-R; MBI; ad hoc	Professional profiles vary according to stress levels and work rewards, indirectly affecting the quality of patient care
de Almeida [61]	2023	Correlational: resilience, social support, socio-demographic variables, working hours, overall health rating	Health professionals (medicine, nursing, psychology)	Quantitative and qualitative	Semi-structured interview; RSA; ad hoc	Social support benefits physical and mental health
Weiss [62]	2024	Predictor: resilience; outcome: burnout, job performance	Pharmacy professionals	Quantitative	BRS; CBI; ad hoc	Resilience negatively predicts burnout

**Table 4:** Quantitative summary of selected studies.

Author (Year)	Country/Income Level	N ^1^ (Professional Profile)	Instruments	Estimators	Key Associations
Alameddine (2022) [50]	Lebanon/upper–middle-income	459 (pharmacists)	CD-RISC; CBI; ad hoc	CD-RISC: M = 68 (SD = 13.37); MBI: M = 56.51 (SD = 16.68)	Lower levels of resilience are associated with higher levels of burnout (β = 0.489; 95% CI, 0.282–0.849, *p* = 0.011)
Alwhaibi (2022) [51]	Saudi Arabia/high-income	139 (health professionals in hospital)	PRIME-MD; MBI; ad hoc	MBI: EE ^2^ M = 31.60 (SD = 15.10); DP ^3^ M = 16.20 (SD = 9.70); PA ^4^ M = 31.50 (SD = 12.80); PRIME MD: depression (61.20%), non-depression (38.80%)	Participants with depression were significantly more likely to present high overall burnout compared to those without depression (20% vs. 16.90%; *p* < 0.001).
Alyahya and AboGazalah (2021) [52]	Saudi Arabia/high-income	275 (health professionals in primary care centers)	PSS; MSPSS	Not reported	Social support is negatively associated with stress (β = −0.15; 95% CI, −0.149–−0.032, *p* < 0.01)
Ayuso-Murillo (2020) [53]	Spain/high-income	197 (nursing professionals in public hospitals)	16PF-5	Anxiety: M = 6.38 (SD = 1.85); warmth: M = 5.58 (SD = 1.62); socially bold: M = 5.60 (SD = 1.74); open to change: M = 5.62 (SD = 1.40)	Anxiety is associated with warmth (t = 2.66, *p* > 0.0001), socially bold (t = −3.17, *p* < 0.001) and open to change (t = −5.81, *p* < 0.0001)
Delafontaine (2024) [54]	Switzerland/high-income	109 (oncology and palliative care health professionals)	MBI; CSDS; TIPI	Not reported	Burnout is associated with positive impact on patient relations (β = −0.22; 95% CI, −0.38–−0.06, *p* = 0.007) and agreeableness (β = −0.18% CI, −0.34–−0.01, *p* = 0.03)
Hunt (2019) [55]	Ireland/high-income	117 (health professionals involved in the care of people with cancer)	ProQOL-V; IRI	Not reported; Cronbach’s alpha: compassion satisfaction (0.88), personal distress (0.71), empathic concern (0.78)	Secondary traumatic stress is positively associated with empathic concern (r = 0.27, *p* < 0.006). Compassion satisfaction is negatively associated with personal distress (r = −0.37, *p* < 0.001)
Jackson (2024) [56]	Japan/high-income	936 (critical care health professionals)	Mini-Z; PHQ-9; GAD-7; BRS-J; ad hoc	Not reported	Depression (β = −0.32, 95% CI −0.41–−0.23) and anxiety (β = −0.20, 95% CI 0.29–0.10) decreased resilience
Johnson (2015) [57]	United States/high-income	40 (health professionals)	CESD-10	Pre: CESD-10: M = 15.80 (SD = 5.01)	CESD-10 mean depression scores decreased 63% from 15.80 to 5.81 (*p* ≤ 0.01) in Resilience Training
Maffoni (2022) [58]	Italy/high-income	315 (health professionals involved in neuro-rehabilitation or palliative care)	CD-RISC; MASI-R; MBI; ad hoc	Not reported	Resilience is positively associated with ethical vision of patient care (β = 0.17, 95% CI 0.09–0.31, *p* < 0.05), well-being (β = 0.48, 95% CI 0.18–0.54) and professional self-efficacy (β = 0.54, 95% CI 0.39–0.65, *p* < 0.001)
Mathebula (2022) [59]	South Africa/upper–middle-income	181 (hospital professionals including administrative staff)	SVEST	Professional self-efficacy including administrative staff: M = 2.71 (SD = 0.94)	Adverse events affect professional self-efficacy (M = 2.51, SD = 0.98)
Ridremont (2024) [60]	France/high-income	262 (medicine, nursing and auxiliary nursing professionals dedicated to child and adolescent cancer care)	PCSQ	PCSQ: M = 10.30 (SD = 1.70)	Significant differences between profiles on total stress score (F = 22.05, *p* < 0.001), stress related to working conditions (W = 31.46, *p* < 0.001), stress related to relationships with patients and families (F = 4.25, *p* < 0.01), stress related to confrontation with suffering and death (F = 3.30, *p* < 0.01), and stress related to relationships with colleagues and superiors (F = 8.59, *p* < 0.001).
de Almeida (2023) [61]	Portugal/high-income	271 (medicine, nursing and psychology)	RSA	RSA: M = 178.17 (SD = 22.44)	Medical doctors and psychologists present the highest levels in total resilience (*p* = 0.018)
Weiss (2024) [62]	United States/high-income	942 (pharmacy professionals)	BRS; CBI	BRS: M = 3.60 (SD = 0.71); CBI: M = 3.22 (SD = 0.92)	Resilience significantly predicted both burnout (β = −0.701, *p* < 0.001) and job performance (β = 0.35, *p* < 0.001)

^1^ N = number of participants; ^2^ EE = emotional exhaustion; ^3^ DP = depersonalization; ^4^ PA = personal accomplishment.

## Data Availability

No new data were created or analyzed in this study.
